# Clinical Scoring Systems for the Assessment of Continence Following Anorectal Malformation Repair: A Comprehensive Review

**DOI:** 10.7759/cureus.89254

**Published:** 2025-08-02

**Authors:** Mohamed S Mohamed, Adel N Assiri, Mohamed Abosheisha, Nazeer Ibraheem, Raouf Ghareb, Mohamed Rashwan, Momen Abdelglil, Ahmed Elkohail, Ali Soffar, Mohamed Mohamed, Abdelmoneim Elshamy, Kareem Abdallah, Mohammed A. E. Ibrahim

**Affiliations:** 1 General Surgery, Hillingdon Hospital, The Hillingdon Hospitals NHS Foundation Trust, London, GBR; 2 General Surgery, Leicester Royal Infirmary Hospital, Leicester, GBR; 3 Urology, New Cross Hospital, The Royal Wolverhampton NHS Trust, Wolverhampton, GBR; 4 General Surgery, Barnet Hospital, Royal Free London NHS Foundation Trust, Barnet, GBR; 5 Critical Care, Hillingdon Hospital, The Hillingdon Hospitals NHS Foundation Trust, London, GBR; 6 Pediatric Surgery, Mansoura University Children's Hospital, Mansoura, EGY; 7 Orthopedics and Trauma, Princess Royal University Hospital, King's College Hospital NHS Foundation Trust, London, GBR; 8 Radiology, Kettering General Hospital, Kettering, GBR; 9 General Surgery, Zagazig University Hospital, Zagazig, EGY; 10 Pediatric Surgery, Manchester University NHS Foundation Trust, Manchester, GBR

**Keywords:** anorectal malformations, clinical scoring systems, continence assessment, fecal continence, fecal incontinence, kelly score, pena score, rintala score

## Abstract

Despite advancements in surgical techniques and improved survival rates for anorectal malformation (ARM) patients, the lack of a standardized way for assessing bowel function and quality of life remains a challenge. Variability in scoring outcomes exists between systems due to inconsistencies in criteria and precise definitions, such as soiling, constipation, and continence. While older tools lack proper validation, newer scales, including the Rintala and Baylor Continence Scale (BCS), offer more structured assessment. There is also a growing acceptance of the need to incorporate quality of life measures, as ARM patients often encounter social and psychological challenges beyond physical symptoms. Multidisciplinary follow-up and age-adjusted evaluations are recommended to enhance long-term outcomes.

## Introduction and background

Anorectal malformation (ARM) is commonly associated with numerous challenges in defecation, even after successful surgical intervention. Consequently, precise measurement of fecal continence (FC) is of paramount importance [1]. 

Despite substantial attention given to the long-term outcomes of patients undergoing ARM repairs over the past few years, the findings continue to be subject to debate. The absence of standardized evaluation tools for assessing functional outcomes has led to a substantial body of literature that lacks the necessary comparability [2]. Several scores for assessing functional capacity in ARM patients have been utilized, but they cannot be compared across different outcomes due to inconsistencies in terminology. No study has been conducted to assess the accuracy of each score for identifying functional capacity issues [3-5].

Currently, there is a lack of international consensus regarding follow-up protocols, objective assessment of ongoing issues, and well-structured transition pathways for children with ARM. Notably, approximately 90% of surgeons do not employ objective, validated assessment tools to monitor bowel and urinary functions, quality of life, or psychosocial concerns. Consequently, it presents a significant challenge to accurately quantify long-term improvements in continence as children develop [6].

Clinical evaluations are frequently subjective and susceptible to observer bias, particularly from treating surgeons. Pediatric surgeons necessitate validated scales and scores to reliably assess patient conditions and functional outcomes. Despite numerous decades of debate, no scoring systems for ARM patients have undergone standardized validation to guarantee reproducibility, validity, or responsiveness [7].

The absence of standardized assessment methodologies hinders the conduct of extensive multicenter research on ARM management, resulting in a scarcity of studies that explore long-term outcomes. Even when scoring systems are employed, there exists a lack of a universally accepted tool or consensus regarding the adjustment of assessments for patients based on their age [8].

## Review

Methodology

A comprehensive literature review was conducted through a systematic literature analysis of the studies included across several databases, including PubMed, Scopus, Web of Science, and Google Scholar. This review covered relevant topic-related publications from the inception of each database until June 2025, resulting in a total of 78 articles. The search utilized a combination of controlled vocabulary (e.g., MeSH terms) and free-text keywords. Key search terms included: "anorectal malformation", "fecal continence", "scoring systems", "continence assessment", "Rintala score", "Kelly score", "Krickenbeck classification", "scoring postoperative results of anorectal malformation", and "pediatric incontinence scales".

Boolean operators (“AND,” “OR”) were applied to refine the results. Reference lists of all relevant full-text articles were manually screened to identify additional studies of interest. The inclusion criteria for this review encompassed peer-reviewed articles written in English that evaluated or compared continence scoring systems in pediatric or adult patients following ARM repair. Eligible studies included reviews, original research articles, and consensus statements that detailed the development, application, or validation of such scoring systems. Studies were excluded if they did not focus on ARM-related continence assessment, were limited to abstracts, letters to the editor, or conference proceedings without full-text availability, or were published in languages other than English. 

Relevant data were extracted and systematically organized for each continence scoring system based on key parameters, including the year of development and author(s), the specific scoring criteria and domains assessed, and the applicability to different patient subgroups such as age or type of ARM. Additionally, the validation status of each tool, covering aspects like internal consistency, reproducibility, and responsiveness, was evaluated. The strengths and limitations of each scoring system were also analyzed to determine their relevance and utility in contemporary clinical and research practice.

Although no formal risk-of-bias tool was applied, included studies were critically appraised based on study design, sample size, methodological clarity, and relevance to the review objective. Special attention was given to studies that provided direct comparisons between different scoring systems or validated them using objective measures such as manometry or quality of life indices. 

Historical review

Several scoring systems have been developed to assess continence following surgery for ARMs in children. The Scott questionnaire, the first continence-scoring questionnaire, was introduced in 1960 and was based on the author’s personal experience. Subsequently, numerous questionnaires have been developed by various medical professionals to address the continence challenges that arise after the repair of ARMs. However, none of these questionnaires has been adequately validated, which explains why no single scoring system has garnered global consensus [9].

An international survey of pediatric colorectal surgeons revealed that 90% do not employ a validated objective tool to monitor postoperative progress in patients with ARM. Furthermore, 81% indicated that the utilization of such a tool would be advantageous [6]. Pediatric surgeons underscore the significance of a standardized and structured approach to long-term follow-up and management to guarantee optimal care and facilitate a seamless transition to adult healthcare providers [2].

Clinical scoring systems for the assessment of continence following ARM repair

Scott Score (1960)

In 1960, Scott devised a scoring system to evaluate continence, categorizing it as “good,” “fair,” or “poor.” “Good” continence is characterized by regular defecation with occasional soiling. “Fair” continence encompasses regular defecation or constipation accompanied by occasional incontinence and soreness. “Poor” continence is defined by frequent bowel movements, persistent soiling, and the absence of sphincter tone [7].

The score had not been adequately validated, and key terms such as “constipation” and “puborectalis sphincter pressure” lacked precise definitions. Despite these limitations, the score was utilized and adapted in an initial study involving children with ARM [10].

Kelly Continence Scoring System (1972)

**Figure 1:**
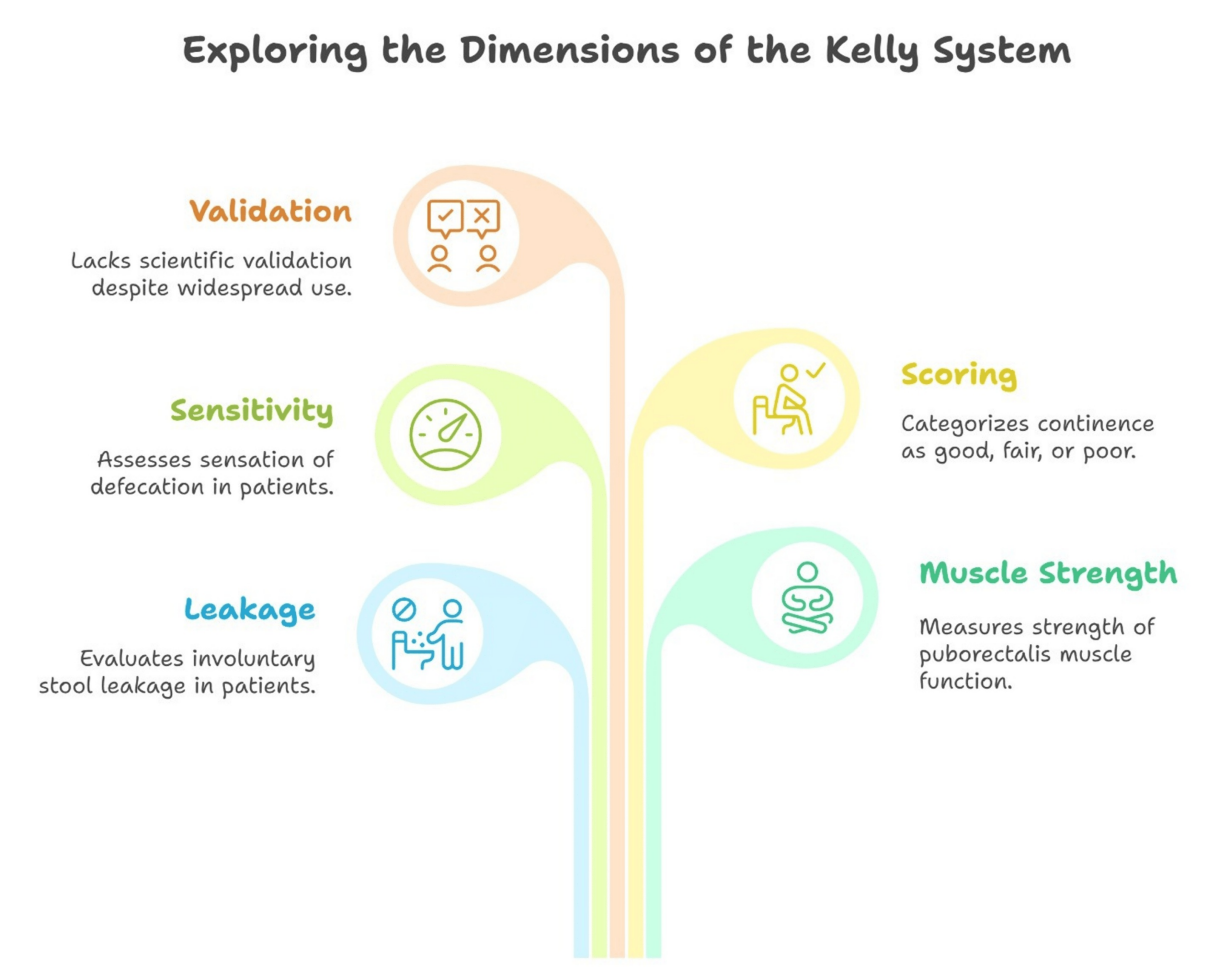
Dimensions of the Kelly scoring system This figure was created by the article's author, Momen Abdelglil, summarizing the dimensions of the Kelly system according to references [3,11].

The Kelly system evaluates continence in patients with ARM by assessing leakage, muscle strength, and sensitivity. It considers factors such as staining, accidental defecation, puborectalis muscle function, and the sensation of defecation [3]. The scoring system categorizes continence as “good” (5-6 points), “fair” (3-4 points), or “poor” (2 points). This assessment aids in determining the functional outcome and potential for treatment improvement (Figure 1) [11].

Although the Kelly score lacks scientific validation, it has gained widespread popularity and is likely the most employed method for assessing fecal incontinence in contemporary practice. It is frequently compared to more objective methodologies, including manometry, electromyography, and assessments of quality of life [12,13]. However, Bischoff et al. (2016) asserted that the sphincter squeeze parameter in the Kelly score is inherently subjective [14].

The Kelly score was used as a quantitative tool to evaluate postoperative FC in patients undergoing laparoscopically assisted anorectal pull-through (LAARP) and posterior sagittal anorectoplasty (PSARP) for high- and intermediate-type ARM [11,15,16].

Holschneider and Modified Holschneider Score (1974)

Holschneider suggested a clinical scoring system to evaluate seven dimensions: fecal frequency, stool consistency, soiling, rectal sensation, stool retention, discrimination, and therapeutic needs. Each parameter is rated from 0 to 2, commensurate with the severity of dysfunction, with precise definitions for factors such as fecal frequency and warning time. The cumulative score categorizes individuals as "continent" (10-14), "fair" (5-9), or "incontinent" (0-4) [17,18]. The author subsequently acknowledged the overemphasis on rectoanal sensitivity parameters. Consequently, they revised the score by reducing the clinical parameters and incorporating manometric data [19].

*Japanese Study Group of Anorectal Anomalies Score (*JSGA) *(1982)*

The JSGA score is a clinical instrument employed to evaluate FC in patients with ARM. Higher scores are assigned to reflect better functional outcomes. Notably, the system evaluates both incontinence and constipation, providing a comprehensive assessment of postoperative outcomes in patients with ARM [17]. The JSGA system assigns scores ranging from 0 to 8 across four fundamental aspects: the urge to defecate (scored from 0 to 2), the severity of constipation (scored from 0 to 4), the frequency of incontinence (scored from 0 to 4), and the frequency of soiling (scored from 0 to 2). 

This system was employed in a study to compare the outcomes of LAARP and PSARP at the ages of four, eight, and 12 years. Despite its utility, no substantial differences were observed in continence outcomes between the respective groups [20].

Templeton and Ditesheim Scoring System (1987)

Ditesheim and Templeton devised a qualitative assessment system for evaluating FC. This system assigns numerical scores based on various factors, including the individual’s awareness of impending bowel movements, unintentional defecation, the necessity of additional undergarments or incontinence pads, social difficulties arising from odor, physical activity restrictions, and the presence of skin rashes. Each criterion is rated from 0 to 1, with a cumulative score of 4 to 5 indicating satisfactory continence, 2 to 3.5 representing moderate continence, and 0 to 1.5 indicating poor continence [21-23].

Wingspread Score (1988)

The Wingspread score is a qualitative score that assesses continence by categorizing it into four main levels: “clean,” “staining,” “intermittent fecal soiling,” and “constant fecal soiling.” It also includes subcategories based on the need for occasional or continuous therapy. This tool has gained significant usage in recent years, particularly considering other associated anomalies [24].

*Peña* *Score (1995)*

Peña proposed a comprehensive evaluation method for assessing long-term outcomes in patients undergoing posterior sagittal anorectoplasty. The study analyzed 387 out of 792 cases and focused on four key parameters: voluntary bowel movements, soiling, constipation, and urinary incontinence. Voluntary bowel movements were assessed based on the patient’s ability to sense, communicate, and control defecation. Soiling was graded from occasional to socially problematic leakage. Constipation was categorized as ranging from dietary management to the need for enemas. Urinary incontinence was evaluated from mild dribbling to complete incontinence [25].

Rintala Score (1997)

There are seven important factors for this scoring system, each rated from 0 to 3. However, the frequency of defecation is scored between 1 and 2, with the maximum achievable bowel function score being 20. A score of 18 or above is considered normal. Patients who score between 7 and 11 points are classified as having "fair" outcomes and experience intermittent daily soiling or staining. Those with "poor" outcomes score between 6 and 9 points, need daily enemas due to severe constipation, or experience constant soiling [26,27].

The Rintala scoring system has gained significant prominence as one of the most extensively utilized and trending scoring systems in recent years, particularly in scholarly investigations pertaining to ARMs [28]. It is relatively objective, does not require physical examination, and is suitable for follow-up evaluations via telephone or online questionnaires. A study revealed a positive correlation between Rintala scores and improved psychosocial well-being and overall quality of life among patients [29].

The Rintala score is also considered a validated score. It was utilized in a study to evaluate bowel function in adults born with ARM or Hirschsprung disease (HD). Data from 133 participants indicated significantly lower bowel function and quality of life scores, particularly in mental health patients compared to healthy individuals [30,31]. Additionally, the Rintala score has been used in various recent studies to assess continence after surgery for ARMs [32].

Kiesewetter and Chang Scoring System (1997)

Kiesewetter and Chang categorized continence into three levels: “good,” which refers to being predominantly continent with infrequent soiling during instances of diarrhea or physical strain, with an average anal resting pressure value of 57.92 ± 8.57 cm H_2_O; “fair,” which describes occasional soiling or staining with normal stool consistency while achieving socially acceptable continence; and “poor,” which denotes severe incontinence with sporadic control or the requirement of a permanent colostomy. For both fair and poor, the average anal resting pressure is recorded as 32 ± 12.83 cm H_2_O [33].

Pediatric Incontinence/Constipation Score (PICS) (2003)

PICS is a validated 13-question instrument designed to assess bowel incontinence and constipation in children aged five and above. It generates separate scores for incontinence (maximum 32 points) and constipation (maximum 29 points), with higher scores indicating improved bowel function. The questionnaire evaluates defecation frequency, stool consistency, urge control, and symptoms such as pain and straining. Developed through rigorous statistical methodologies, PICS is instrumental in monitoring bowel health, monitoring progress over time, and evaluating treatment or surgical outcomes [2,22,34].

Krickenbeck Score (2005)

The Krickenbeck classification assesses the functional outcomes of patients with ARM following definitive surgery, particularly focusing on voluntary bowel movements, soiling, and constipation. Furthermore, the Krickenbeck scoring system is extensively employed to evaluate functional outcomes in children with HD following surgical intervention [5].

The Krickenbeck system stands out as the most objective scoring method, as it meticulously evaluates two distinct factors: the absence of involuntary bowel movements and the presence of fecal matter. Nevertheless, its primary limitation lies in the inability to reassess patients following interventions. To mitigate this challenge, a modification to the Krickenbeck system is imperative, enabling its application to patients who successfully achieve voluntary bowel movements through the utilization of laxatives or constipating agents [14].

Baylor Continence Scale (BCS) (2007)

BCS is a 23-item questionnaire meticulously designed to assess bowel function based on clinical observations. This observer-independent instrument can be completed by children or their parents without necessitating a clinical examination. The scores span from 2 to 84, with lower scores indicating improved social continence. The BCS has been validated for differentiating between normal children and those experiencing bowel function disorders. In a study, the BCS was employed to evaluate the continence outcomes of children undergoing treatment for low-type ARM [35].

Analysis of different scores according to the literature

**Figure 2:**
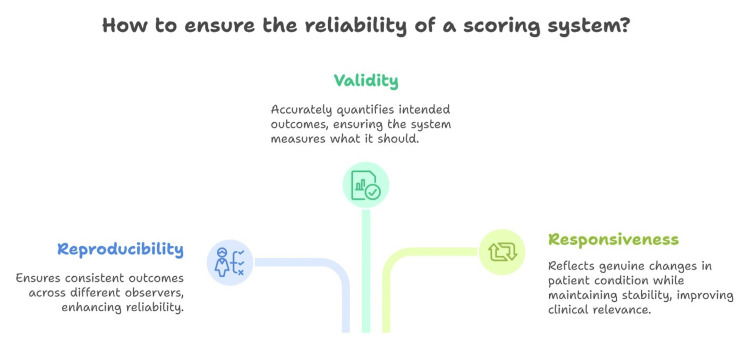
Reproducibility, validity, and responsiveness of scoring systems This figure was created by the article's author, Momen Abdelglil, summarizing the validation process of scoring systems according to the reference [36].

To guarantee reliability, a scoring system must exhibit reproducibility (consistent outcomes across diverse observers), validity (accurately quantifying the intended outcomes), and responsiveness (reflecting genuine alterations in the patient’s condition while maintaining stability otherwise). These attributes should be established through a structured approach rather than relying solely on clinical intuition. Nevertheless, numerous scoring systems primarily serve descriptive purposes and lack standardized validation for these criteria. Additionally, inconsistencies in defining endpoints such as constipation and intermittent defecation complicate the evaluation of outcomes (Figure 2) [36].

Limited research has examined the comparative efficacy of distinct scoring systems within the same study cohort. Ong and Beasley conducted a comparative analysis of four scoring methodologies, employing the Kelly, Templeton and Ditesheim, Kiesewetter and Chang, and Wingspread systems. The results demonstrated substantial variability, with the Templeton score indicating a superior level of FC compared to the other three systems [37,38].

The discrepancies in FC scoring outcomes across studies are primarily attributed to the utilization of diverse scoring systems, each assessing bowel function through distinct criteria and response options. The Templeton and Ditesheim system represents a quantitative method comparable to other well-established systems such as Kelly, Kiesewetter, and Wingspread. Over time, various scoring systems (e.g., Holschneider, Rintala, Krickenbeck, and Peña) have been developed, yet their results exhibit substantial variability. For instance, the questionnaires employed by Krickenbeck and Peña tend to yield lower scores in low-type ARMs compared to high-type, in contrast to the Holschneider and Rintala systems. This underscores the necessity of a standardized and unified bowel function assessment instrument for patients with ARM [21].

The majority of documented scoring systems require interpretation due to the inclusion of subjective parameters such as “sensation of rectal fullness,” “sphincter contraction,” or “anal shape.” Additionally, these systems often focus on multiple factors unrelated to fecal incontinence, including bowel movement frequency, rectal prolapse, abdominal discomfort, hemorrhoids, urinary leakage, diarrhoea, and constipation. Numerous scoring systems exhibit imperfections due to their subjective nature, introducing bias and interpretation. Conversely, those focused on quality of life fail to address the fundamental issue of bowel control. The Krickenbeck score emerges as the most pertinent and objective approach for assessing bowel control in pediatric patients. Its utility may further be enhanced when adapted to evaluate patients’ post-medical intervention [14].

The Rintala score is a known established score. It was used by Wehrli et al. (2023) to evaluate bowel function in adults with ARM [30,31,39]. The BCS has also undergone a validation process [35].

Ochi et al. (2012) conducted a comparative study of the Kelly, JSGA, and Holschneider scoring systems for evaluating postoperative FC in 86 patients with ARM. The results indicated that these protocols yielded inconsistent outcomes, particularly in male high-type cases, with some assessments differing by one or two outcome levels. The JSGA protocol exhibited the highest variability due to its primary focus on constipation and incontinence. The study underscored the limitations of existing scoring systems, emphasizing the necessity of standardized, quality-of-life-oriented assessment tools. Furthermore, it suggested that simpler qualitative classifications may provide a more accurate representation of patients’ postoperative status [17].

**Figure 3:**
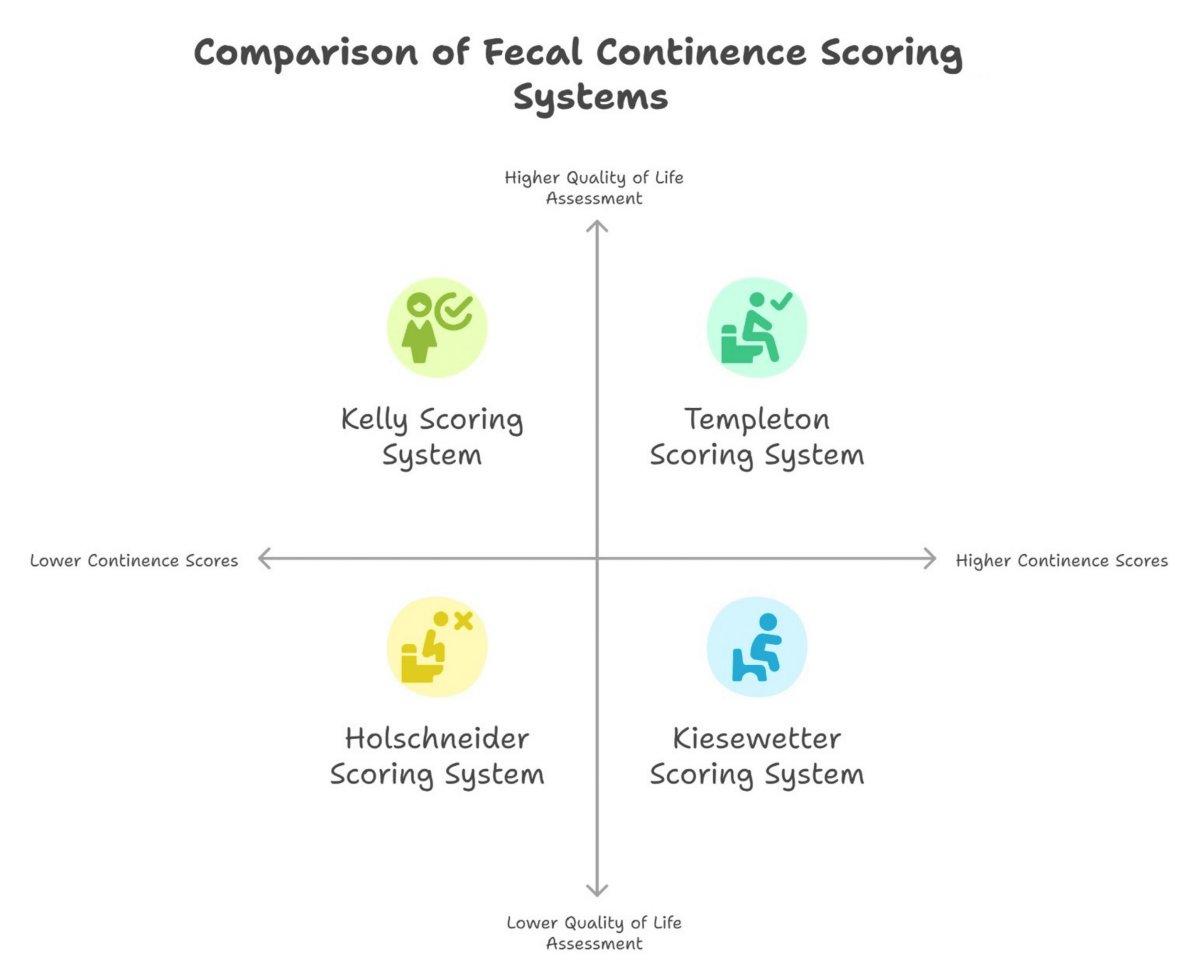
Comparison of fecal continence scoring systems This figure was created by the article's author, Momen Abdelglil, summarizing the fecal continence scoring systems for postoperative follow-up of children with anorectal malformations according to references [17,35,40].

The primary reason for the observed disparities across diverse studies lies in the utilization of disparate FC scoring systems. These systems evaluate bowel function from distinct perspectives and offer varying response options. Notably, the Templeton and Ditesheim scoring system is a quantitative method that has demonstrated comparability with other systems, including the Kelly, Kiesewetter, and Wingspread systems. However, it tends to assign higher continence scores compared to systems such as Holschneider, Rintala, Krickenbeck, and Peña, which are also employed to analyze bowel function but exhibit significant variations in their outcomes [21]. One of the notable strengths of the Templeton scoring system is its inclusion of items that assess the quality of life in children with ARM. These items encompass various aspects, including school attendance, social relationships, and physical abilities (Figure 3) [40].

Shaari et al. (2024) conducted an evaluation of the usability and consistency of four bowel function scoring systems in assessing postoperative outcomes for patients with ARM. The study found that all systems are broadly comparable, albeit with varying strengths. The Kelly score is distinguished by its ease of understanding and inter-rater consistency, while the Krickenbeck score exhibits the strongest correlation with anomaly types. Notably, both the Kelly and Holschneider scores are deficient in measures for constipation, thereby limiting their comprehensiveness [41].

Brisighelli et al. (2018) discovered that the overall scores from these systems exhibited comparable patterns, with discernible variations when examining specific types of ARMs. The Krickenbeck and Peña questionnaires, which are highly similar, generally yielded lower scores for ARMs with favourable prognoses and higher scores for those with unfavourable prognoses in comparison to Holschneider and Rintala. The study underscores the intricate nature of comparing outcomes due to the progression of distinct classifications and scoring systems, emphasizing the imperative for a universally recognized scoring system to facilitate standardized assessments across diverse patient cohorts and treatment regimens [42].

In a seminal review published in 1990, Ong and Beasley comprehensively analysed FC scoring systems, elucidating their distinct strengths and limitations. The Kelly method stands out for its objective and quantitative assessments, achieved through digital examination. Conversely, the Templeton system prioritizes the quality of life aspect, albeit without incorporating physical evaluations. The Kiesewetter criteria categorize continence levels based on societal acceptability, albeit without providing precise definitions. The Wingspread scheme offers detailed assessments of stool accumulation and therapy dependency, making it impractical for comparative purposes. Each of these systems strikes a unique balance between objectivity, simplicity, and comprehensiveness [37].

The Kelly scoring system primarily focuses on accidents and sphincter function, neglecting constipation. Conversely, the JSGA score comprehensively evaluates both incontinence and constipation, with lower scores indicating improved outcomes. The Holschneider score encompasses a broader spectrum of symptoms, often resulting in elevated scores for constipated patients. Notably, Scott et al. and Schärli overlooked constipation, while Rintala and Lindahl included it but provided limited attention, potentially leading to an inadequate assessment of postoperative FC [17,43-45]. Divergences in scoring systems may contribute to variations in outcomes. In 2002, Holschneider et al. proposed the replacement of scoring systems with a classification system based on the treatment required for postoperative FC [9,17].

A recent study has identified Holschneider’s questionnaire (HQ) and Rintala’s questionnaire (RQ) as the most effective clinical scoring systems for monitoring patient follow-up after ARM repair. The study analyzed anorectal manometry data from 23 patients and found statistically significant correlations between anal resting pressure and HQ, as well as between the area under the curve (AUC) of the maximum voluntary squeeze pressure-time graph and both HQ and RQ. Furthermore, significant differences in HQ and RQ scores were observed between high-type and low-type ARMs. In contrast, Peña’s questionnaire and Krickenbeck’s questionnaire were recommended for guiding bowel management programs rather than for follow-up monitoring [18].

## Conclusions

The assessment of FC following ARM repair exhibits inconsistencies due to the absence of a universally accepted scoring methodology. Although newer scales, such as the Rintala and BCS, provide structured evaluations, standardization and validation remain imperative.
